# Effects of Resveratrol, Lovastatin and the mTOR-Inhibitor RAD-001 on Insulin-Induced Genomic Damage In Vitro

**DOI:** 10.3390/molecules22122207

**Published:** 2017-12-12

**Authors:** Eman Awad, Eman M. Othman, Helga Stopper

**Affiliations:** 1 Institute of Pharmacology and Toxicology, University of Würzburg, 97078 Würzburg, Germany; eman.awad@uni-wuerzburg.de (E.A.); eman@toxi.uni-wuerzburg.de (E.M.O.); 2 Department of Analytical Chemistry, Faculty of Pharmacy, University of Minia, Minia 11432, Egypt

**Keywords:** insulin, resveratrol, lovastatin, mTOR-inhibitor RAD-001, genomic damage

## Abstract

Diabetes mellitus (DM) is one of the major current health problems due to lifestyle changes. Before diagnosis and in the early years of disease, insulin blood levels are elevated. However, insulin generates low levels of reactive oxygen species (ROS) which are integral to the regulation of a variety of intracellular signaling pathways, but excess levels of insulin may also lead to DNA oxidation and DNA damage. Three pharmaceutical compounds, resveratrol, lovastatin and the mTOR-inhibitor RAD-001, were investigated due to their known beneficial effects. They showed protective properties against genotoxic damage and significantly reduced ROS after in vitro treatment of cultured cells with insulin. Therefore, the selected pharmaceuticals may be attractive candidates to be considered for support of DM therapy.

## 1. Introduction

Diabetes Mellitus (DM) is a class of chronic metabolic disorder occurring as insulin dependent type I or non-insulin-dependent type II according to the definition of the World Health Organization (WHO) [1]. DM is one of the major health problems throughout the world in the 21st century, where the risk of diabetes might increase due to lifestyle changes [2,3]. The number of patients with DM is expected to increase up to 366 million by 2030 [4]. Patients with type II diabetes have many severe complications, such as kidney and heart disease and retinopathy with increased risk of morbidity and mortality [5,6,7].

Additionally, insulin resistance and increased oxidative stress have been observed in type II diabetic patients [8,9,10,11]. Increased production of ROS and altered cellular redox status are related to many diseases including hyperinsulinemia [12]. Many prospective studies suggest that hyperinsulinemia may be an important risk factor for different types of cancer [13,14,15,16,17,18,19,20,21], with the kidney being one of the main targets [22,23]. In a prediabetes status and during the first years of disease, blood insulin levels are elevated in DM type II, insulin generates low levels of reactive oxygen species which are integral to the regulation of a variety of intracellular signaling pathways [24,25]. Insulin actions are initiated by activation of the insulin receptor (IR) with intrinsic tyrosine kinase activity, thus triggering the activation of different intracellular protein substrates such as insulin receptor substrates (IRSs) [26]. This stimulates the regulatory subunit of the phosphatidylinositol-3-kinase (PI3K) which activates the Protein Kinase B (AKT) pathway that is responsible for many important physiological metabolic actions of insulin such as glycogen, lipids and protein synthesis. Recently, our group described that nanomolar concentrations of insulin, which are less than 10-fold higher than pathophysiological levels achieved in vivo, can induce reactive oxygen species in cultured cells in vitro, leading to DNA damage. In kidney cells, mitochondria and NOX4 are involved in reactive oxygen species production and are downstream of the activation of the PI3K/AKT pathway [27].

In recent decades, studies suggested that using natural products could modify the insulin signaling pathway; such compounds may affect the influence of insulin signaling outcomes with low cost and limited or no side effects [28,29]. Here we selected three particularly interesting natural and pharmacological compounds with a potential to interfere with the insulin action cascade, namely resveratrol [30,31], lovastatin [32,33], and the mTORC1 inhibitor RAD-001 [34,35]. Inhibition of insulin mediated genotoxicity may be relevant for cancer protection of patients with elevated insulin levels and the mTOR pathway has not been investigated for this purpose before. In addition, the protective effect of resveratrol and lovastatin against insulin have not been shown regarding genomic damage. In the normal non-transformed rat kidney cells that we used to represent the kidney as one of the major cancer target tissues in diabetes type II, we show here that several principles of molecular activity can be applied to ameliorate the genotoxicity of insulin.

## 2. Results

### 2.1. Vitality Test for Mammalian Cultured Cells (NRK)

To investigate the effects of resveratrol, lovastatin and RAD-001 on insulin, we performed vitality tests for cultured normal rat kidney (NRK) cells. The NRK cells were treated with resveratrol (1 μM), lovastatin (10 nM) or RAD-001 (5 nM) for 15 min before the addition of insulin (10 and 100 nM) for 2 h. Then the percentages of viable and non-viable cells were quantified. Under the tested conditions, cells did not show a significant decrease in viability after 2 h treatment for resveratrol, lovastatin, RAD-001, insulin and their combinations compared with the respective controls except for the combination of RAD-001 with insulin. However, with values of still more than 95% viable cells, it was in the similar range as all other treatments and the statistical significance is not biologically meaningful in this case (Table 1).

### 2.2. The Intrinsic Antioxidant Capacity

The intrinsic antioxidant capacity of resveratrol, lovastatin and RAD-001 were assessed in a cell free system by ferric reducing antioxidant power (FRAP) assay using tempol (50 μM) as a positive control. The results showed that lovastatin and RAD-001 exhibited no intrinsic antioxidant activity (no significant increase over control), while resveratrol at 1 μM yielded a significant increase (equivalent to 3.58 ± 0.19 μM Fe-reduction capacity) in absorption over control.

### 2.3. Effect of Resveratrol, Lovastatin and mTORC1 Inhibitor RAD-001 on Genomic Damage

**Figure 1:**
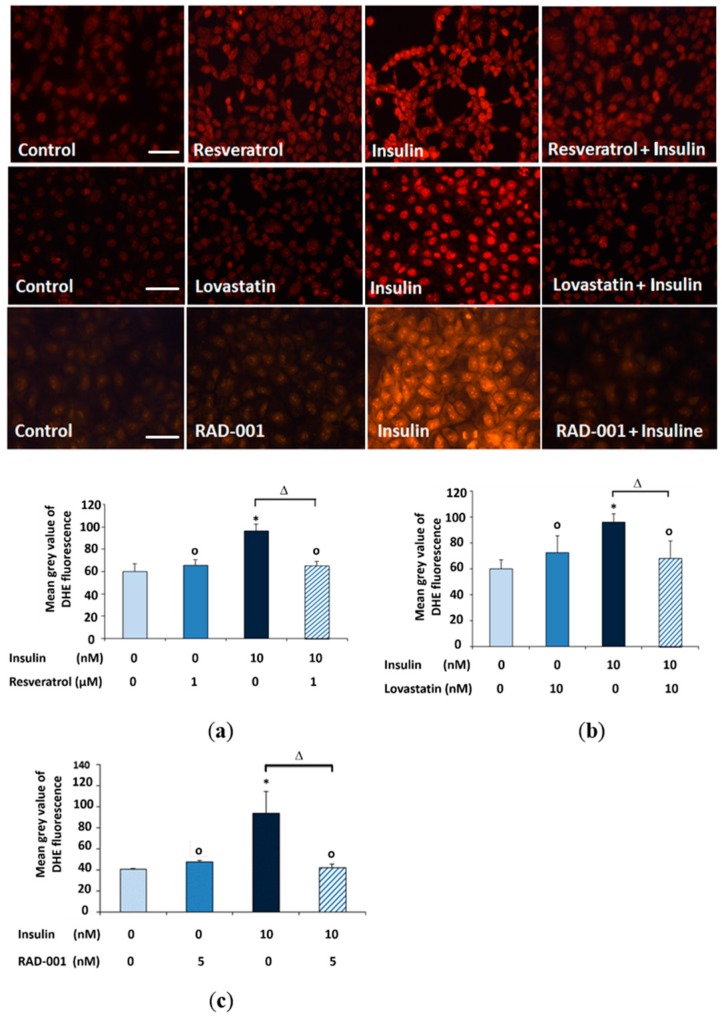
Microscopic detection of superoxide formation using the dye DHE in NRK cells treated for 15 min with (**a**) 1 μM resveratrol; (**b**) 10 nM lovastatin and (**c**) 5 nM RAD-001 then addition of 10 nM insulin for 30 min in the presence of DHE. Quantification of DHE fluorescence was done by measuring the mean grey value of 200 cells using image j software. (*) Significantly different from control, (Δ) significantly different from insulin and (o) not significantly different from control. Scale bars 50 μm.

In order to investigate the potential antioxidant activity of resveratrol, lovastatin or mTOR inhibitor RAD-001 in a cellular system, the cells were treated with insulin for 30 min and reactive oxygen species were detected. Prior addition of resveratrol, lovastatin or RAD-001 to the cells reduced the insulin mediated ROS production (Figure 1).

**Figure 2:**
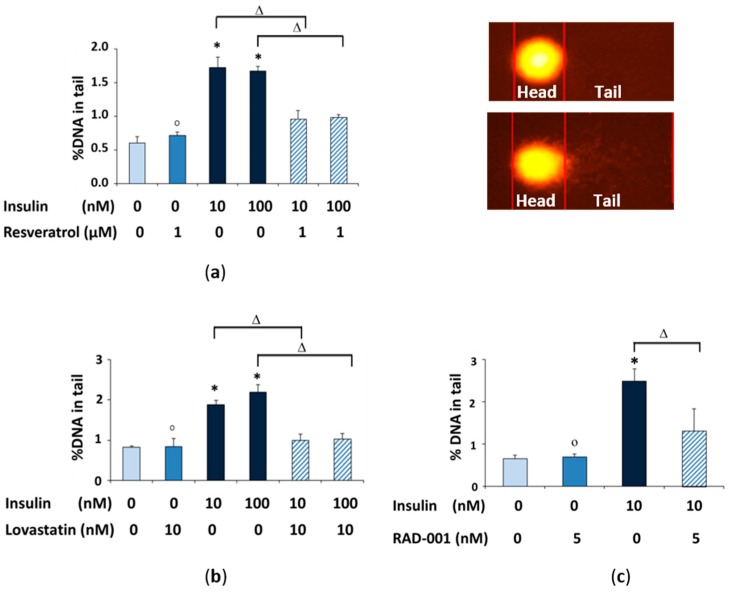
DNA damage (% DNA in tail) measured with the comet assay after treatment of NRK cells with (**a**) 1 μM resveratrol; (**b**) 10 nM lovastatin and (**c**) 5 nM RAD-001 for 15 min with different concentrations of insulin for 2 h. (*) Significantly different from control, (Δ) significantly different from insulin and (o) not-significantly different from control.

To examine the potential protective activity of resveratrol, lovastatin and RAD-001 against DNA damage, comet assay and micronucleus frequency test were performed. At first, we used 10 and 100 nM concentrations of insulin (resveratrol and lovastatin). We expected to have additional damage using higher than the physiological concentration, but the results showed no significant difference. Therefore, we applied 10 nM insulin for combination with RAD-100 because it is much closer to physiological levels and therefore more relevant. Treatment of the cells with insulin for 2 h yielded a significant induction of DNA damage in the comet assay compared to the control cells, while the cells reacted with significant reduction of DNA damage if the three compounds were added 15 min before the 2 h insulin treatment (Figure 2).

**Figure 3:**
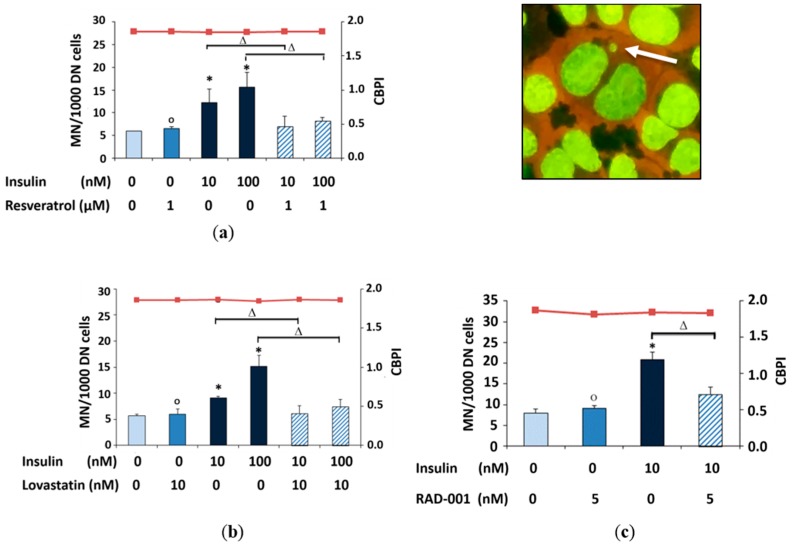
Micronucleus frequency (MN/1000 DN cells; DN = double nucleated cells) and proliferation index (CBPI) in NRK cells treated with (**a**) 1 μM resveratrol; (**b**) 10 nM lovastatin and (**c**) 5 nM RAD-001 for15 min. before the addition of insulin for 4 h. Harvest was after an additional 22 h expression time. (*) Significantly different from control, (Δ) significantly different from insulin and (o) not significantly different from control. The cytokinesis-block proliferation index [CBPI] is represented as red line ( ■ ) and shown on the second *Y*-axis.

The micronucleus induction assay was carried out by treating the NRK cells with 10 nM and 100 nM insulin for 4 h treatment time. Moreover, the addition of these compounds 15 min before the addition of insulin decreased the formation of micronuclei induced by insulin (Figure 3). Additionally, the cell proliferation (CBPI) was unaffected.

### 2.4. Phosphorylation of AKT

**Figure 4:**
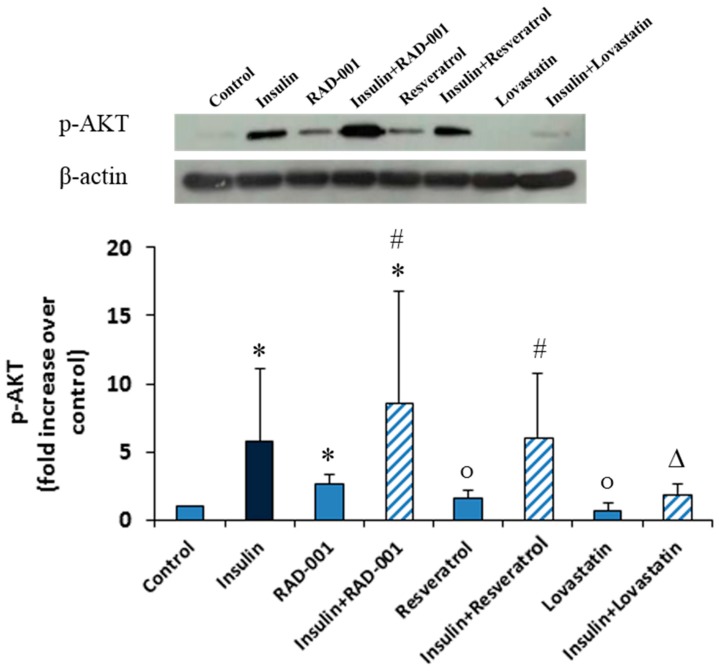
p-AKT level in NRK cells treated with 10 nM insulin, 5 nM RAD-001, 1 μM resveratrol and 10 nM lovastatin and their combination with insulin for 2 h and analyzed by Western blotting. Blots from 3 independent experiments were used for quantification. (*) Significantly different from control, (Δ) significantly different from insulin, (o) not significantly different from control and (#) not significantly different from insulin.

The quantification of the western blot analysis of phosphorylated AKT (p-AKT) in NRK cells showed that a significant increase in the amount of p-AKT protein was observed after insulin treatment compared to the control. RAD-001 enhanced significantly the amount of p-AKT, while resveratrol showed small enhancement of the amount of p-AKT and lovastatin alone exerted no effect. In combination with insulin, lovastatin prevented the elevation of p-AKT after insulin treatment, but resveratrol did not exert any influence and RAD-001 even increased the amount of detectable p-AKT after insulin treatment but not significantly compared with insulin alone (Figure 4).

## 3. Discussion

The chosen compounds resveratrol, lovastatin and RAD-001 have an impact on insulin signaling via specific mechanisms as proposed for each of these compounds [30,31,32,33,34]. Resveratrol is thought to reduce the activation of AKT, and the mammalian target of rapamycin (mTOR) in a dose and time dependent manner in particular with high concentration and long exposure time [36] and stimulated glucose uptake in skeletal muscle and adipose tissue [37,38]. Lovastatin has an ability to inhibit the function of ligand-induced receptor activation and downstream signaling through the PI3K/AKT pathway and also by inhibition of NOX4 [39]. mTORC1 inhibitors such as RAD-001 can play a key role in the regulation of the PI3K/AKT/mTOR pathway via attenuation of mTORC1 downstream targets.

Resveratrol is a polyphenolic compound that is mainly produced in the skin of red grapes, has been reported to enhance health, improve lifespan by 60% in short-lived fish [40] and mimic caloric restriction as a sirtuin activator [41]. Additionally, resveratrol has been studied at a wide range of concentrations by Bhat et al. due to its pharmacological properties [42]. However, resveratrol also showed bifunctional effects through different actions including both antioxidant and pro-oxidant effects depending on the dose and treatment time [43]. Thus, the present study investigated the protective effects of resveratrol on DNA damage which had been enhanced by insulin treatment in kidney cells in vitro. Our finding showed that resveratrol treatment reduced oxidative stress, DNA damage and micronucleus formation in NRK cells. Moreover, the western blot analysis showed that the phosphorylation level of AKT activated by insulin was not affected by resveratrol. However, resveratrol was able to reduce Fe^3+^ to Fe^2+^ in a cell free system. Our findings are in agreement with Santos et al. who reported that the protective effect of resveratrol under conditions of oxidative stress induced by hydrogen peroxide in C6 glioma cells was able to prevent oxidative damage to cellular DNA, after a short exposure time of resveratrol (0.5 h; 10–250 μM of resveratrol) [44].

However, long time treatment with resveratrol (>6–48 h) induced a slight DNA damage in time and dose-dependent [44]. Additionally, Aydın et al. showed that in resveratrol treated septic rats, the oxidative DNA damage in liver and kidney cells was inhibited significantly due to elevated levels of reduced glutathione (GSH), superoxide dismutase (SOD) and glutathione peroxidase (GP_X_) activities [45]. Overall, our results suggest that the effect of resveratrol under our treatment conditions had no effect on the insulin signaling pathways but that it acts as a protecting agent against DNA damage due to its antioxidant properties.

Lovastatin belongs to the class of 3-hydroxy-3-methylglutaryl coenzyme-A (HMG-CoA) reductase inhibitors. It is also a naturally occurring compound which can be found in food such as oyster mushrooms [46] and red yeast rice [47]. It is used mainly to reduce cholesterol in order to decrease the risk of cardiovascular disease in people who suffer from hypercholesterolemia [48]. Our results showed that lovastatin did not exhibit intrinsic antioxidant activity in FRAP assay but attenuated the ROS overproduction induced by insulin in kidney cells as well as protected cells from DNA damage and micronucleus formation after insulin treatment. Western blot analysis confirmed that p-AKT expression enhanced by insulin was significantly suppressed by lovastatin. Our results are in agreement with Mcguire T. F. et al. who showed that lovastatin disrupts the association of PI3K with IR/IRS-1 complex in HIR rat-1 fibroblasts [32]. Thus, it seems conceivable that lovastatin caused its protective effects via a signaling-mediated activity.

Everolimus (RAD-001) is a semi-synthetic mTOR inhibitor targeting specifically the raptor/mTOR complex 1 (mTORC1). mTOR, via other target including S6K and 4E-BP1, is a widely expressed key regulator for several functions such as; cell growth, proliferation, survival and autophagy [49]. It can be activated by different stimuli including nutrients and growth factors [49]. In addition, mTORC1 through S6K induces negative feedback loops that suppress the activation of insulin-AKT signaling pathway. However, the inhibition of the negative feedback mediates AKT phosphorylation [50].

As we previously described, the stimulation of NRK cells by insulin immediately upregulates intracellular kinase signaling involved in the activation of the PI3K/AKT signaling pathway, followed by activation of NADPH oxidase and mitochondria and resulting in the generation of ROS production [27]. Published studies showed that overactivation of mTOR caused an increase in mitochondrial biogenesis, accumulation of reactive oxygen species (ROS), and thereby, more DNA damage [51]. Additionally, down-regulating of mitophagy by activation of mTOR might be involved in the induction of ROS through accumulation of damaged mitochondria [52,53]. Mitochondria are among the main cellular sources of ROS production through complex III due to the electron leakage from complex I [54]. Thus, the inhibition of raptor/mTOR complex by RAD-001 may affect mitochondrial activity through the reduction of mitochondrial complex I activity [55].

In agreement with this idea, we found no increase in ROS production after addition of RAD-001. Furthermore, the pretreatment of the cells with the mTORC1 inhibitor blocked the ROS formation stimulated by insulin under the tested conditions. The pretreatment of the kidney cells with RAD-001 before the addition of insulin also reduced the DNA and genomic damage induced by insulin, although it did not protect the cells completely. Additionally, western blot analysis showed that RAD-001 enhanced the amount of p-AKT as did the combination of RAD-001 and insulin.

However, this enhancement in p-AKT resulting from the combination was not significant compared to insulin. Our findings are in agreement with Nacarelli et al. who showed that pretreatment of human diploid fibroblasts with rapamycin reduced the level of mitochondrial ROS generation induced by ethidium bromide and increased the phosphorylation of AKT [56]. Moreover, Miwa et al. suggested that reduction of mTOR signaling pathway by rapamycin might improve the function of telomerase protein TERT in mitochondria which is important to suppress mitochondrial ROS [57]. Overall, disruption of the mTORC1 complex by RAD-001 lowers oxidative stress and thereby, limits the damage to cellular components and that could be due to effects on mitochondria via insulin signaling pathway.

## 4. Materials and Methods

### 4.1. Chemicals

Human insulin, lovastatin and resveratrol were purchased from Sigma-Aldrich (St. Louis, MO, USA, or Munich, Germany). RAD-001 was purchased from MedChemTronica (Stockholm, Sweden). Gel Red and Gel Green were purchased from Biotrend (Köln, Germany). Cell culture media and reagents were obtained from PAA Laboratories GmbH (Pasching, Austria) and Invitrogen Life Technologies (Carlsbad, CA, USA, or Darmstadt, Germany). Anti-AKT (pS473) antibody (22650) was purchased from Rockland (Gilbertsville, PA, USA). Anti ß-actin antibody (T6199) was purchased from Sigma-Aldrich (Taufkirchen, Germany).

### 4.2. Cell Culture

Normal rat kidney epithelial cells (NRK) were obtained from European Collection of Cell Culture (ECACC, Salisbury, UK) and grown at 37 °C with 5% CO_2_ in DMEM medium (4.5 g/L glucose) supplemented with 10% fetal calf serum, 2 mM l-glutamine, 1% non-essential amino acids and 1% antibiotics (50 U/mL penicillin and 50 mg/mL streptomycin). They were subcultured twice per week.

### 4.3. Vitality Test

Cells were seeded one day before the experiment in a control medium. After treatment of the cells with lovastatin, resveratrol or RAD-001 for 15 min followed by the addition of insulin for 2 h, cells were harvested and 70 μL of the cell suspension was stained with 30 μL Gel RedBiotrend (Köln, Germany) staining solution. Twenty microliters of this mixture were applied to the slide, and the fractions of green and red cells in a total of 200 cells were counted at a 500-fold magnification with a fluorescence microscope.

### 4.4. Ferric Reduction Antioxidant Power (FRAP)

Resveratrol, lovastatin and RAD-001 were assessed for antioxidant activity in a cell free system using the ferric reducing antioxidant of plasma (FRAP) method [58] which determines the reduction of a ferric tripyridyltriazine complex to its colored form (Fe^3+^ to Fe^2+^). Briefly, 20 μL of sample was added to 180 μL of water. Next, 600 μL of the FRAP reagent (1:1:10 mixture of 10 mM ferric tripyridyltriazine, 20 mM ferric chloride and 300 mM acetate buffer) was added and the absorption at 593 nm was measured after 3 min. The results were quantified according to a standard curve produced using different concentration of ferrous sulfate.

### 4.5. Microscopic Analysis of the Formation of Reactive Oxygen Species (ROS)

Evaluation of the formation of ROS was performed using the cell-permeable fluorogenic probe DHE. One day before the experiment, 2 × 10^5^ cells were seeded in 24-mm cover slips in 6-well plates in 3 mL medium; after treatment of the NRK cells with the selected compounds and 10 μM DHE, the cells were incubated in the dark at 37 °C for 30 min. After that, ROS production was detected after washing with 500 μL PBS, the cover slips were mounted on a slide and observed under an Eclipse 55i microscope (Nikon GmbH, Düsseldorf, Germany) and a Fluoro Pro MP 5000 camera (Intas Science Imaging Instruments GmbH, Göttingen, Germany) at 200-fold magnification. All DHE staining images were taken using the same exposure time. Quantification was carried out by measuring gray values of 200 cells per treatment using ImageJ 1.40 g (http://rsb.info.nih.gov/ij/).

### 4.6. Comet Assay

The alkaline version of the comet assay detects single- and double-strand breaks as well as alkali-labile lesions on an individual cell basis as a standard test for genotoxicity [59]. Briefly, NRK cells were treated with resveratrol, lovastatin or RAD-001 (1 μM, 10 nM or 5 nM, respectively) for 15 min, then insulin (10 or 100 nM) for 2 h. After the cells were harvested, 20 μL of the treated cells suspension were mixed with 180 μL of 0.5% low-melting agarose and added to fully frosted slides that had been covered with a bottom layer of 1% normal melting point agarose. The slides were incubated in lysis solution (2.5 M NaCl, 0.1 M EDTA, 0.01 M Tris, and 10-g/L *N*-lauroylsarcosine sodium adjusted to pH 10 with NaOH) with 1% Triton X-100 and 10% dimethyl sulfoxide at 4 °C. After at least 1 h, the slides were washed and then placed in the electrophoresis solution (300 mM NaOH and 1 mM EDTA, pH 13) for 20 min. Then the electrophoresis was conducted for 20 min at 25 V (1.1 V/cm) and 300 mA. The slides were neutralized in 0.4 M Tris buffer (pH 7.5) and then dehydrated in methanol for 5 min at −20 °C. Then, the slides were dried and stored at room temperature. After staining of each slide with 20 μL of Gel Red/diazabicyclo octane (DABCO) solution for detection of DNA, images of 100 randomly selected cells (50 per replicate slide) for each sample were analyzed with a fluorescence microscope (Labophot 2; Nikon GmbH, Düsseldorf, Germany) at 200-fold magnification using image analysis software (Komet 5; BFi OPTiLAS, Gröbenzell, Germany). The percentage of DNA in the tail was used to quantify DNA migration.

### 4.7. Micronucleus Frequency Test

Micronuclei are small chromatin containing structures in the cytoplasm of cells, which represent a subtype of chromosomal aberrations. 3 × 10^6^ Cells/mL were incubated with resveratrol, lovastatin and RAD-001 for 15 min and followed by addition of insulin for 4 h in 3 mL medium. After that, the medium was removed and replaced by fresh culture medium with cytochalasin B (3 μg/mL) after washing with PBS. After a further 20–22 h, cells were harvested, brought onto glass slides by cytospin centrifugation, and fixed in methanol (−20 °C) for at least two hours. Before counting, cells were stained for 3 min with Gel Green (10 μL stock solution in 990 μL distilled water), washed twice with PBS buffer, and mounted for microscopy. Due to the cytokinesis inhibitor cytochalasin B, mitosis results in double-nucleated cells. From each of the 2 slides, 1000 such double nucleated cells were evaluated for micronuclei, and the average was calculated. For substance combinations, concentrations which were described as effective in the literature and had been found not toxic in preliminary experiments were applied. Additionally, the cytokinesis-block proliferation index (CBPI) was calculated as an assessment of potential cytostatic effects from 1000 cells per sample using the following formula: CBPI = (number of mononucleated cells + 2 × number of double nucleated cells + 3 × number of multinucleated cells)/(mononucleated + double nucleated + multinucleated cells).

### 4.8. Western Blot Analysis

After treatment of cells with insulin, resveratrol (1 μM), lovastatin (10 nM) or RAD-001 (5 nM) and the combinations for 2 h, cells were harvested and lysed in Ripa buffer, which contained freshly added protease inhibitor cocktail (PIC), sodium orthovanadate and sodium fluoride to inhibit protease and phosphatase activity. The homogenization process was facilitated by mechanical disruption of the cell membranes and the obtained suspension was then centrifuged at 14,000 rpm for 30 min at 4 °C. The protein containing supernatant was transferred to a clean tube and the concentration of protein in this solution was determined using Bradford’s method. Generally, 30 μg of protein per sample was loaded on acrylamide gel. After electrophoresis the gel was blotted on PVDF membrane. The membrane was blocked overnight in either 5% bovine serum albumin for p-AKT, 5% nonfat milk powder for β-actin in TBS-T buffer (5 mM TRIS, 150 mM NaCl, 0.05% Tween-20) and then incubated with primary antibody (p-AKT (1:2000) and β-actin (1:5000)). After that the excess of primary antibody was washed off for 3 × 10 min with TBS-T buffer, then the horse radish peroxidase (HRP) conjugated secondary antibody was added followed by washing 3 × 10 min with TBS-T buffer again. After incubation with HRP substrate, the membrane was exposed to an X-ray sensitive film and the film was developed afterward.

### 4.9. Statistics

In vitro data are from three independent experiments ± standard deviation (SD). Statistical analysis was performed with IBM SPSS version 22 software. The Mann-Whitney U-test was used to determine the significance between two treatments. All results were considered significant if *p* ≤ 0.05.

## 5. Conclusions

Resveratrol, lovastatin and RAD-001 showed protective properties and suppressed the genomic damage mediated by insulin in kidney cells in vitro, which is here described for the first time. A combination of these compounds with insulin may be of potential relevance for the support of T2DM treatment and help in the prevention of co-morbid diseases such as cancer. It is relevant that this can be achieved by several principles of molecular activity, which may enable greater chances for development of intervention strategies for patients.

## Figures and Tables

**Table 1:** Viability of normal rat kidney (NRK) cells treated with resveratrol, lovastatin, RAD-001, insulin and combinations with insulin.

	Treatment	Viability (%)
**Group I**	Control	95.33 ± 0.58
	Resveratrol	96.12 ± 1.53
	10 nM Insulin	97.00 ± 1.00
	100 nM Insulin	95.83 ± 1.15
	Resveratrol + 10 nM Insulin	97.12 ± 1.53
	Resveratrol + 100 nM Insulin	97.67 ± 1.15
**Group II**	Control	95.17 ± 1.53
	Lovastatin	95.17 ± 0.58
	10 nM Insulin	97.38 ± 1.15
	100 nM Insulin	96.34 ± 1.53
	Lovastatin + 10 nM Insulin	97.12 ± 1.15
	Lovastatin + 100 nM Insulin	95.67 ± 1.53
**Group III**	Control	98.00 ± 1.00
	RAD-001	97.67 ± 0.83
	10 nM Insulin	98.17 ± 1.53
	RAD-001 + 10 nM Insulin	95.67 ± 1.53
